# Freeze-FRESH: A 3D Printing Technique to Produce Biomaterial Scaffolds with Hierarchical Porosity

**DOI:** 10.3390/ma13020354

**Published:** 2020-01-12

**Authors:** Zi Wang, Stephen J. Florczyk

**Affiliations:** 1 Department of Materials Science & Engineering, University of Central Florida, Orlando, FL 32816, USA; 2 Burnett School of Biomedical Sciences, University of Central Florida, Orlando, FL 32827, USA

**Keywords:** freeze-casting, additive manufacturing, cell-material interaction, tumor microenvironment, regenerative medicine

## Abstract

Tissues are organized in hierarchical structures comprised of nanoscale, microscale, and macroscale features. Incorporating hierarchical structures into biomaterial scaffolds may enable better resemblance of native tissue structures and improve cell interaction, but it is challenging to produce such scaffolds using a single conventional scaffold production technique. We developed the Freeze-FRESH (FF) technique that combines FRESH 3D printing (3DP) and freeze-casting to produce 3D printed scaffolds with microscale pores in the struts. FF scaffolds were produced by extrusion 3DP using a support bath at room temperature, followed by freezing and lyophilization, then the FF scaffolds were recovered from the bath and crosslinked. The FF scaffolds had a hierarchical pore structure from the combination of microscale pores throughout the scaffold struts and macroscale pores in the printed design, while control scaffolds had only macroscale pores. FF scaffolds frozen at −20 °C and −80 °C had similar pore sizes, due to freezing in the support bath. The −20 °C and −80 °C FF scaffolds had porous struts with 63.55% ± 2.59% and 56.72% ± 13.17% strut porosity, respectively, while control scaffolds had a strut porosity of 3.15% ± 2.20%. The −20 °C and −80 °C FF scaffolds were softer than control scaffolds: they had pore wall stiffness of 0.17 ± 0.06 MPa and 0.23 ± 0.05 MPa, respectively, compared to 1.31 ± 0.39 MPa for the control. The FF scaffolds had increased resilience in bending compared with control. FF scaffolds supported MDA-MB-231 cell growth and had significantly greater cell numbers than control scaffolds. Cells formed clusters on the porous struts of FF scaffolds and had similar morphologies as the freeze cast scaffolds. The FF technique successfully introduced microscale porosity into the 3DP scaffold struts to produce hierarchical pore structures that enhanced MDA-MB-231 growth.

## 1. Introduction

Tissues have hierarchical structures comprised of components that span multiple length scales. Bone is an example of a tissue with hierarchical structure, as it has features that range from nanoscale bone mineral crystals to macroscopic features in cortical bone [1]. Biomaterial scaffolds are produced in many different formats using a variety of methods in an attempt to replicate tissue structures. The scaffold structure and stiffness influence the cell response through cell adhesion, cell–matrix interaction, and mechanotransduction [2,3]. However, many biomaterial scaffolds have features at a single length scale, not hierarchical structures consisting of features on multiple length scales. Scaffolds with hierarchical structures may better resemble biological tissues and have been demonstrated to enhance cell function [4,5,6].

Several 3D printing (3DP) techniques, including extrusion printing, fused deposition modeling, stereolithography, inkjet printing, and selective laser sintering, have been used to produce polymer, ceramic, and metal biomaterial scaffolds with designed structures [7,8]. While these 3DP techniques enable production of complex structures, the 3DP scaffolds typically consist of smooth, solid struts with the scaffold porosity resulting from the print design. These 3DP structures may limit cell interaction as the cells may sense the scaffold struts as a flat surface if the strut diameter is large enough [9]. Extrusion 3DP uses aqueous polymer solutions to produce scaffolds, however, solidification mechanisms, such as ionic crosslinking and UV curing, are required to prevent the structure from collapsing due to gravity [10]. Supporting baths have also been used for extrusion 3DP to encapsulate extruded polymer in situ until stabilized [11,12,13,14]. A 3DP technique called freeform reversible embedding of suspended hydrogels (FRESH) utilized a gelatin support bath to encapsulate printed hydrogel structures [13,14], while a similar 3DP technique utilized a Carbopol ETD 2020 polymer support bath [11].

Freeze-casting is a common method to produce 3D porous polymer and ceramic biomaterial scaffolds, where a solution is cast in molds, frozen, and lyophilized to produce porous scaffolds with an interconnected porous network [15,16,17]. Ice crystals nucleate and grow throughout the solution during freezing, excluding the material and creating material-rich and material-poor regions, with the material-rich regions yielding the final scaffold structure [16,17]. The ice is removed by freeze drying and the polymer scaffold is stabilized by neutralization or crosslinking. The freeze cast (FC) scaffold pore size can be adjusted by altering the solution concentration and varying the freezing temperature, with lower temperatures producing finer pore sizes [16,17]. FC scaffolds have been produced with polymers and ceramics for a variety of tissue engineering [18,19,20,21] and tumor microenvironment [22,23,24,25,26] applications. While freeze-casting is a versatile method to produce scaffolds, the overall scaffold structure is limited by the mold. The mold limitations of FC scaffold design have been addressed by cryogenic printing, a technique that combines 3DP with freeze-casting to incorporate porous features on 3DP scaffold struts [27,28,29]. Cryogenic printing extrudes a polymer solution that is frozen upon deposition on to a cold stage or into a cold chamber or cold bath. Liquid nitrogen has been used as a cold bath and to cool the stage, while dry ice and isopropanol has been used to cool the stage. Cryogenic printing has several limitations, including: (1) the solution deposition rate must be tuned to allow freezing shortly after deposition, as complications occur if the solution freezes too quickly or too slowly; (2); the printed structures have non-uniform porosity and the pore sizes in the struts may vary by scaffold layer; (3) extra equipment is required for the cold stage, chamber, or bath; and (4) the build height is restricted to the dimensions of the cold chamber or stage.

Herein, we report the Freeze-FRESH (FF) 3DP technique. The FF technique combines FRESH 3DP and freeze-casting to produce 3DP scaffolds with microscale pores in the printed struts. The scaffold is printed at room temperature in the FF technique and the entire scaffold is frozen within the support bath, unlike cryogenic printing, where the scaffold is frozen as it is printed on the cold stage. The FF scaffold materials properties, including pore morphology, pore size, porosity, shrinkage, and stiffness, were characterized and compared to smooth (FRESH) scaffolds. Human MDA-MB-231 breast cancer cells were cultured on the FF scaffolds for 7 d to analyze the effect of the strut porosity on cell morphology and cell growth.

## 2. Materials and Methods

### 2.1. Preparation of Gelatin Support Bath

The gelatin slurry support bath was prepared by adapting the published FRESH method [13]. Briefly, 10 g of gelatin (Type A, Sigma-Aldrich, St. Louis, MO, USA) and 0.4 g of calcium chloride (CaCl_2_) (Sigma-Aldrich, St. Louis, MO, USA) were added to 250 mL of deionized (DI) water at 45 °C in a 500 mL glass Mason jar (Ball Inc., Broomfield, CO, USA) stirred until dissolved, then refrigerated overnight at 4 °C. Next, 250 mL of 0.16 w/v% CaCl_2_ solution at 4 °C was added to the jar and the contents were blended at pulse speed for 90 s using a blender (Oster Heritage Blend 400, Sunbeam Products Inc., Boca Raton, FL, USA). The blended gelatin slurry was transferred to several 50 mL conical tubes and diluted with cold CaCl_2_ solution. The gelatin slurry was washed by vigorously vortexing the mixture, centrifuging at 3700 × g for 2 min, then aspirating the supernatant. The slurry was washed three times with cold CaCl_2_ solution to completely remove soluble gelatin. Gelatin slurry was stored in suspension with CaCl_2_ solution at 4 °C until printing. To prepare the support bath for printing, the gelatin was centrifuged at 225 × g for 5 min to compact the gelatin slurry, the supernatant was aspirated, then the slurry was poured into 35 mm Petri dishes and covered with Kimwipes (Kimberly-Clark, Irving, TX, USA) to remove any excess fluid.

### 2.2. Preparation of Inks for Printing

A solution of 4 w/v% sodium alginate (FMC BioPolymer, Philadelphia, PA, USA) and 0.4 w/v% hyaluronic acid (HA, hyaluronic acid sodium salt from Streptococcus equi, Sigma-Aldrich, St. Louis, MO, USA) was prepared in DI water. HA was added to the alginate solution to increase solution viscosity to improve printing and to improve cell adhesion since alginate is relatively bioinert and has poor cell adhesion [30]. The solution was mixed in a Thinky mixer (ARM-300, Thinky, Laguna Hills, CA, USA) at 2000 rpm for 5 min twice until fully dissolved. RGD-alginate was prepared by functionalization with RGD peptide (Abcam, Cambridge, MA, USA) using standard carbodiimide chemistry following a published method [31]. Briefly, 10 mg of the RGD peptide was dissolved in 0.1 M PBS and added to 50 mL 4 w/v% alginate solution. 4 mM 1-ethyl-dimethylaminopropyl carbodiimide (EDC, Sigma-Aldrich, St. Louis, MO, USA) and 10 mM N-hydroxysulfosuccinimide (sulfo-NHS, Sigma-Aldrich, St. Louis, MO, USA) were added sequentially to alginate solution to initiate conjugation. The mixture reacted for 20 h with continuous stirring and was stopped by adding 5 mM hydroxyl amine hydrochloride (Alfa Aesar, Haverhill, MA, USA). The solution was loaded into dialysis membranes (MWCO 12000, Spectra/Por^®^, New Brunswick, NJ, USA) and dialyzed for 3 d in DI water with DI water changed daily. The dialyzed RGD-alginate was frozen at −20 °C overnight and lyophilized for 24 h, then stored at 4 °C until use. A 4 w/v% RGD-alginate solution was prepared in DI water by mixing with the Thinky mixer at 2000 rpm for 5 min twice until dissolved. The 4 w/v% RGD-alginate solution was combined with the 4 w/v% alginate-HA solution at equal volume and mixed with Thinky mixer at 2000 rpm for 5 min twice to produce the RGD-alginate ink. The inks were dyed with food coloring (McCormick, Baltimore, MD, USA) to visualize the scaffold during printing.

### 2.3. Freeze-FRESH Printing Method

A 3D cuboid model with dimensions of 20 mm × 20 mm × 6 mm (length × width × height) was created using SolidWorks (Dassault Systèmes, Vélizy-Villacoublay, France) and converted to STL file format using MeshLab (http://meshlab.sourceforge.net/). The STL file was loaded into Repetier-Host and sliced using the Slic3r plugin into a solid honeycomb model with 25% infill density and 0.5 mm layer height. A scaled version of the cuboid was prepared at 10 mm × 10 mm × 4 mm with the same 25% infill density slicing setting to obtain the scaled honeycomb prints. The G-code for the honeycomb model STL file was exported from Repetier-Host and loaded into the operating interface of Biobots 1 3D printer (Allevi Inc., Philadelphia, PA, USA) for FF printing. Alginate ink was drawn into a 10 mL Luer-Lock syringe (BD, Franklin Lakes, NJ, USA) and a 27 gauge (0.21 mm inner diameter) 0.5-inch stainless steel flat tipped needle (Jensen Global, Inc., Santa Barbara, CA, USA) was used as the nozzle for printing. The syringe was connected to the air compressor and inserted into the extruder on the 3D printer, then the z-axis calibration was performed. A 35 mm Petri dish containing gelatin support bath was placed on the printing platform. The extrusion printing was performed at room temperature of 22 ± 1 °C with the extruder traveling speed at 8 mm/s and air pressure at 25 psi.

After printing, the prints were frozen within the gelatin bath in a −20 °C or −80 °C freezer overnight (>12 h). Frozen samples were lyophilized with a freeze drier (Virtis Freezemobile, SP Scientific, Warminster, PA, USA) for 24 h. The scaffolds were recovered from the gelatin support bath by soaking the support bath and printed scaffold in 0.2 M CaCl_2_ solution at 37 °C in a beaker on a hot plate. The gelatin melted and the calcium ions in solution crosslinked the printed structures during scaffold recovery. Smooth scaffold controls were prepared with the same printing process and recovery method, but without the freezing step. Control FC alginate scaffolds were prepared by casting the alginate ink into 24-well plate molds, freezing overnight at −20 °C, and lyophilizing to form cylindrical porous scaffolds. FC scaffolds were cut into 2 mm thick discs and processed in the same manner as the 3DP scaffolds.

Smooth, FF, and FC scaffolds were immersed in 0.2 M CaCl_2_ solution and crosslinked under vacuum for 30 min. The scaffolds were washed with DI water three times after crosslinking and scaffolds for cell culture were sterilized in 70% ethanol for 10 min under vacuum, with the ethanol solution changed after 5 min. The sterile alginate scaffolds were transferred to a biosafety cabinet and washed with sterile Dulbecco’s phosphate buffered saline (D-PBS, FisherScientific, Hampton, NH, USA) three times to remove the ethanol. Scaffolds were soaked in sterile 0.2 M CaCl_2_ for 5 min to further crosslink the structure. The scaffolds were transferred to 12-well plates and washed with cell culture media twice, then incubated in media overnight in the incubator at 37 °C.

A human femur model (3dprint.nih.gov) was printed to demonstrate the ability of the FF method to produce complex biological structures. The dimensions of the femur were 44.96 mm × 7.95 mm × 6.91 mm (l × w × h). The femur STL file was sliced at 25% infill density and with a layer height of 0.5 mm for printing. Femur prints were frozen, freeze dried, and recovered in the same manner as the FF scaffolds. The pore formation in the femur structures was examined using purple food coloring (McCormick, Baltimore, MD, USA) as a 10 v/v% solution in DI water. The smooth and FF femurs were immersed in the dye solution for 60 s, then excess dye solution was wiped away and the femurs were photographed.

### 2.4. Pore Morphology

Pore morphology of the scaffolds was characterized with scanning electron microscopy (SEM) imaging. The scaffolds were recovered, washed with DI water three times, immersed in DI water, frozen at −20 °C, and lyophilized for 24 h to preserve the porous structure for SEM imaging. The prints were sectioned longitudinally and transversely using double edge razor blades. Samples were mounted on stubs, sputter coated with gold, and imaged with a JSM-6480 SEM (JEOL, Tokyo, Japan ).

### 2.5. Measurement of Scaffold Shrinkage, Porosity, and Pore Size

Scaffold shrinkage was calculated as the percent volume change of the recovered scaffolds relative to the volume of the 3D model design. The volume of scaffolds (*n* = 8) was obtained by measuring the dimensions with a digital micrometer (Mitutoyo, Aurora, IL, USA). The percent volume change of FC scaffolds was calculated relative to the volume of the mold, a well in a 24-well size plate.

The porosity measurements were conducted on FF and smooth scaffolds. The dimensions of smooth and FF scaffolds were measured with a digital micrometer to determine the scaffold volume. FC scaffolds were cut into 10 mm × 10 mm × 5 mm (l × w × h) cubes and their dimensions were measured with a digital micrometer to determine the volume. Porosity was measured using a modified liquid displacement method with isopropanol used as the displacement liquid [25,32]. The weight (W_i_) of the dry scaffolds (*n* = 6) was measured using an analytical balance (ML54T, Mettler Toledo, Columbus, OH, USA). All scaffolds from each group were immersed in 15 mL of isopropanol of known density (ρ_i_ = 0.785 g/mL) in a 50 mL tube under vacuum for 1 h, then the saturated scaffolds were weighed (W_f_). Porosity for FC scaffolds was presented as bulk porosity and calculated as a ratio of the volume of solvent within the scaffold pores to the volume of the dry scaffold (Equation (1)). Due to the macroscale porosity (>1 mm diameter) in the smooth and FF scaffolds, the porosity calculation was adjusted to quantify the porosity present on the struts. The volume of the macroscale porosity in the scaffold was calculated by measuring the macroscale pore area on SEM images using ImageJ (1.51, National Institutes of Health (NIH), Bethesda, MD, USA), calculating the volume of the macroscale pores, and multiplying by the number of macroscale pores (8) in the scaffold. The macroscale pore volume was subtracted from the scaffold volume to generate the strut volume. The weight of isopropanol in the macroscale porosity was calculated by multiplying the density of isopropanol with total macroscale pore volume. The final weight was calculated after subtracting the weight of isopropanol in the macroscale porosity. The strut volume and final weight were entered into Equation (1) to yield strut porosity.

(1) $$Porosity=\frac{\left(W_{f}-W_{i}\right)/\rho_{i}}{V_{i}}\;\times \;100\%$$

The pore size measurements were performed with SEM images of the samples. Low magnification SEM images were used to measure the macroscale pore size and higher magnification SEM images were used to measure the microscale pore size. The measurements were calibrated to the image scale and the pore size was calculated assuming circular pores. Five samples were imaged for each group and multiple images were taken to cover each sample to assess the macroscale pore size. The pore diameter for all visible macropores (>100 pores in total for each sample group) was measured with ImageJ (1.51, NIH, Bethesda, MD, USA) to yield an average pore size. The microscale pore size measurements were performed with ImageJ (1.51, NIH, Bethesda, MD, USA) using a total of six SEM images per group with two images from three different samples. Pores in the focal plane were randomly selected and the pore diameter was measured. At least 18 measurements were performed for each image and the average microscale pore size was calculated for each group. A total of 190, 195, and 175 measurements were conducted on −20 °C FF, −80 °C FF, and FC scaffolds, respectively.

### 2.6. Mechanical Characterization

The scaffold compressive modulus was measured in the wet state. The smooth and FF scaffolds were prepared at 20 mm × 20 mm × 6 mm (l × w × h) dimensions. After recovery and crosslinking the average −20 °C and −80 °C FF scaffold dimensions were 16.26 mm × 16.47 mm × 4.35 mm and 16.52 mm × 16.27 mm × 4.16 mm, respectively, and the smooth scaffold dimensions were 13.05 mm × 13.09 mm × 3.76 mm. FC scaffolds were cut into 10 mm × 10 mm × 5 mm (l × w × h) cubes and crosslinked with CaCl_2_ solution before testing. The samples (*n* = 8 per group) were damp with D-PBS during testing and were compressed at a rate of 0.4 mm/min using an AGS-X mechanical tester (Shimadzu, Columbia, MD, USA) with a 500 N load cell. The bulk scaffold stiffness was determined from the slope of linear elastic portion (0%–15% strain) and pore wall stiffness was determined from the slope of ultimate compression portion (60%–80% strain) on the stress–strain curves [33]. The cross-sectional area of the 3D printed scaffolds (smooth and FF) was normalized by the print infill density. Both smooth and FF scaffolds had a 25% infill density, so the measured cross-sectional area was divided by four to normalize the stiffness data.

### 2.7. Cell Culture

GFP-transfected MDA-MB-231 (231) breast cancer cells (GenTarget Inc., San Diego, CA, USA) were expanded to 80% confluency in T75 cell culture flasks with fully supplemented media consisting of Dulbecco’s modified Eagle medium (DMEM, Corning Inc., Corning, NY, USA), 10 v/v% fetal bovine serum (Atlanta Biologicals, Flowery Branch, GA, USA), 1 v/v% penicillin/streptomycin (FisherScientific, Hampton, NH, USA), and 1 v/v% non-essential amino acids (FisherScientific, Hampton, NH, USA). Cells were detached with 0.25 w/v% trypsin-ethylenediaminetetraacetate (EDTA; FisherScientific, Hampton, NH, USA), which was inactivated with fully supplemented media, and centrifuged at 500 × g for 5 min to pellet the cells. The 10 mm × 10 mm × 4 mm 3DP scaffolds, FC scaffolds, poly ε-caprolactone (PCL) scaffolds (Sigma-Aldrich, St. Louis, MO, USA), and 2D wells were seeded with 100,000 cells in 50 μL of media in 12-well plates and incubated for 2 h before adding fully supplemented media. Samples were cultured at 37 °C and 5% CO_2_ in a fully humidified incubator for 7 d with regular media changes every 2 d.

### 2.8. Cellular Analysis 

Cell number was analyzed with Alamar Blue assay at 3 and 7 d. The media was aspirated and scaffolds were washed with D-PBS. A 10 v/v% Alamar Blue solution was prepared by adding Alamar Blue reagent into fully supplemented media and 1.5 mL of 10 v/v% Alamar Blue solution was added to each sample well. Samples were incubated at 37 °C for 2 h, then 300 μL Alamar Blue reagent from each well was transferred in duplicate to a black 96-well plate. Fluorescence measurement was performed using a Cytation5 cell imaging multi-mode plate reader (Biotek, Winooski, VT, USA) at an excitation wavelength of 560 nm and emission wavelength of 590 nm. Cell number was calculated using a standard curve generated from a series of known numbers of cells grown as monolayers. The 231 cell morphologies were observed with fluorescence imaging using a Cytation5 cell imaging multi-mode plate reader.

### 2.9. Statistical Analysis

All data are reported as the mean ± standard deviation (SD). Statistical significance was set at *p* < 0.05 and the results were analyzed using analysis of variance (ANOVA) followed by *t*-tests between paired groups (Microsoft Excel, 16.29.1, Redmond, WA, USA).

## 3. Results and Discussion

### 3.1. FF Method Produces Scaffolds with Hierarchical Porosity

**Figure 1:**
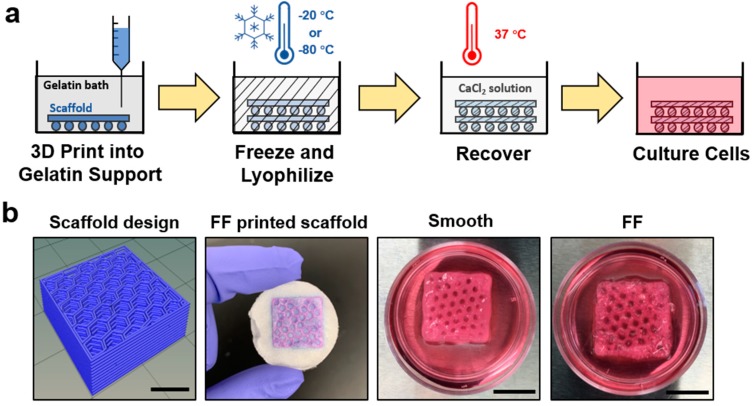
The Freeze-FRESH (FF) technique enables 3D printing of biomaterial scaffolds with hierarchical porosity. (**a**) A schematic illustration of the FF technique. (**b**) Comparison of the scaffold design, the FF scaffold in support bath after freezing and freeze drying, and the smooth and FF 3D printed scaffolds after recovery from the support bath. The recovered scaffolds were immersed in cell culture media. The bubbles on the FF scaffold struts denote porosity on the scaffold struts. Scale bars are 10 mm.

It is difficult to produce biomaterial scaffolds with hierarchical structures using a single common scaffold production technique. The FF method was developed to produce 3DP scaffolds with hierarchical porosity through the macroscale pores in the scaffold design and microscale pores on printed scaffold struts. The FF process is shown schematically in Figure 1a, where polymer scaffolds are extrusion 3D printed into a gelatin support bath, frozen, and lyophilized to produce microscale porosity on scaffold struts. The FF scaffold was recovered from the gelatin support bath and the printed structure was crosslinked. Recovered scaffolds were immersed in cell culture media to evaluate the scaffold stability (Figure 1b). The FF and smooth scaffolds had good shape fidelity after recovery and were stable when soaked in cell culture media for 1 h. The smooth scaffolds had no bubbles present around the struts, while the FF scaffolds had bubbles surrounding the struts, indicating pore formation in the struts.

FF scaffolds were evaluated for scaffold shrinkage, porosity, and pore size in comparison with smooth and FC scaffolds (Table 1). The FF scaffolds were frozen at −20 °C and −80 °C to investigate the influence of freezing temperature on pore size. Both the smooth and FF scaffolds had considerable shrinkage compared to the scaffold design: the smooth scaffolds were 73.02% ± 4.44% smaller and the FF scaffolds frozen at −20 °C and −80 °C were 51.23% ± 6.96% and 53.39% ± 7.87% smaller, respectively. The FC scaffolds were 22.06% ± 1.47% smaller than the mold size. FF scaffolds frozen at −20 °C and −80 °C had strut porosities of 63.55% ± 2.59% and 56.72% ± 13.17%, while smooth scaffolds had a strut porosity of 3.15% ± 2.20%. This indicates that the FF scaffold struts were porous while there were few pores in the smooth scaffold struts. FC scaffolds had the highest porosity of 90.03% ± 3.31%, however this was for the entire structure since it had no macroscale pores.

The smooth and FF scaffolds had greater shrinkage than FC scaffolds. The macroscale porosity in the smooth and FF scaffolds contributed to the greater shrinkage as there is less material present in an equivalent scaffold volume. Freezing limited the scaffold shrinkage, as the smooth scaffolds had greatest shrinkage compared to FF and FC scaffolds. Freezing the polymer solutions alters the polymer orientation as the polymer molecules are rejected by the ice crystals and densified into pore walls [34]. The densified polymer networks in the FF and FC scaffolds limits the motion of polymer molecules compared to the smooth scaffolds, where polymer molecules have spaces to move. This difference in polymer structure between the scaffold types caused less shrinkage for FF and FC scaffolds compared to smooth scaffolds.

**Figure 2:**
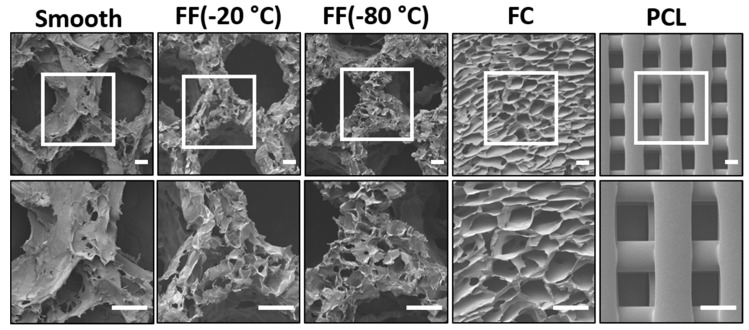
The FF technique produces scaffolds with hierarchical pore structures. Morphological differences in the pore structures of alginate scaffolds: smooth, FF frozen at −20 °C and −80 °C, FC, and PCL 3D printed scaffolds. Scale bars are 300 μm.

The FF scaffold pore structure was characterized with SEM imaging (Figure 2) and pore size measurements (Table 1). FF scaffolds had both macroscale and microscale pores producing a hierarchical pore structure, while there were no microscale pores present in the struts of smooth scaffolds (Figure 2). The smooth and FF scaffolds had similar macroscale pores since they were printed with the same design. The average macroscale pore size in the scaffold design was 3.21 ± 0.14 mm, while the average macroscale pore size for smooth scaffolds was 1.26 ± 0.23 mm and for FF scaffolds frozen at −20 °C and −80 °C was 1.41 ± 0.26 mm and 1.33 ± 0.25 mm, respectively. The −20 °C and −80 °C FF scaffolds had similar pore structures with microscale pores present in the printed struts (Figure 2) with average pore sizes of 307.79 ± 97.12 μm and 269.17 ± 58.39 μm, respectively (Table 1). FC and commercial PCL scaffolds were used as controls for the FF scaffolds. FC scaffolds had microscale pores with an average pore size of 152.42 ± 41.73 μm, while the PCL scaffolds had a crosshatch structure with 300 μm fiber diameter, 300 μm fiber spacing, and smooth strut surfaces.

**Figure 3:**
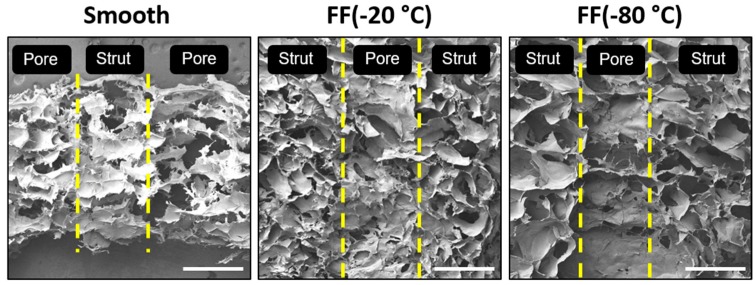
Vertical cross-sections of smooth and FF scaffold struts. Both the −20 °C and −80 °C FF scaffold struts had pores, while the smooth scaffold struts did not. The yellow lines indicate the edges of struts and pores (macroscale pores). Scale bars are 1 mm.

The cross-sections of the smooth and FF scaffold struts were also examined with SEM imaging to determine the pore structure found within the scaffold pores (Figure 3). The strut cross-sections showed that both the −20 °C and −80 °C FF scaffold struts had a porous structure, while the smooth scaffold struts showed few porous features and those present appear to be artifacts of the SEM sample preparation. The pore size of the pores within the struts was similar to the microscale pore sizes reported in Table 1. The vertical cross-sections of the FC scaffolds were not examined in this study, but based on our previous work, we could assume that the FC scaffolds will have a similar pore size in the vertical cross-section as the horizontal cross-section.

The similar pore sizes in −20 °C and −80 °C FF scaffolds could result from the gelatin particles in the support bath altering the freezing process. This could also explain the greater pore size of −20 °C FF scaffolds compared to FC scaffolds that were also frozen at −20 °C. In the freeze-casting process, the mold containing the cast solution is placed on a cold surface and the heat transfer starts from the cold surface, which creates a freezing front that propagates from bottom to the top of the sample [35]. The freezing front velocity influences the scaffold pore structure, as it affects the morphology of the ice crystals that template the scaffold pore structure with colder temperatures producing finer pores [16,17]. Freezing the FF scaffolds within the support bath of fine gelatin particles that have been packed into a bed with little excess liquid provides an atypical environment for freeze-casting. The combination of the fine particles and the dry support bath may have insulated the printed scaffold, altering the freezing behavior. Further investigation is needed to determine how the gelatin support bath affected the freezing behavior of the FF prints.

### 3.2. Characterization of FF Scaffold Mechanical Properties

**Figure 4:**
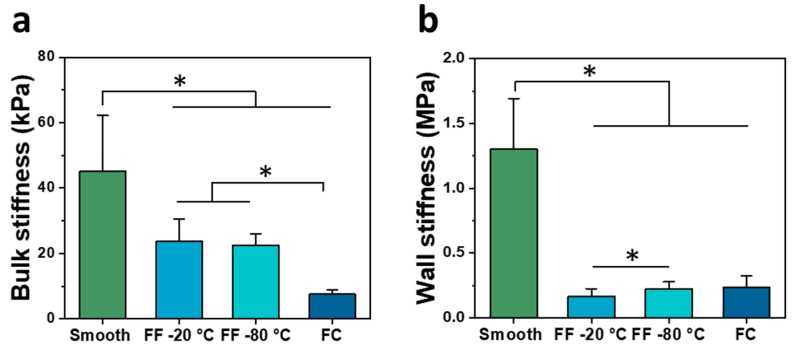
Mechanical properties of FF scaffolds. Bulk (**a**) and wall (**b**) stiffness in wet conditions of smooth, FF frozen at −20 °C and −80 °C, and FC scaffolds. (*) denotes significant difference where *p* < 0.05.

**Figure 5:**
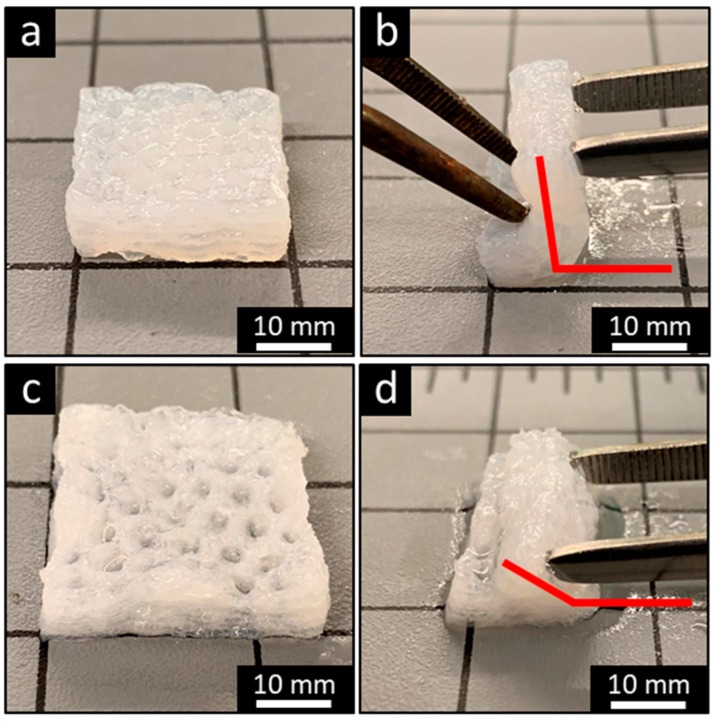
The FF technique promotes scaffold resiliency. Bending of smooth (**a, b**) and −20 °C FF (**c, d**) scaffolds demonstrates that the FF scaffolds have a greater bending angle than smooth scaffolds.

The scaffolds were characterized in compression in wet conditions to determine the bulk stiffness (Figure 4a) and pore wall stiffness (Figure 4b). The smooth scaffolds had the greatest bulk stiffness of 45.19 ± 16.97 kPa, followed by the −20 °C and −80 °C FF scaffolds with the bulk stiffness of 23.75 ± 6.73 kPa and 22.47 ± 3.51 kPa, respectively. The FC scaffolds had the least bulk stiffness of 7.56 ± 1.44 kPa. There were significant differences between all paired groups except for the −20 °C and −80 °C FF scaffolds. The smooth scaffolds had the greatest wall stiffness of 1.31 ± 0.39 MPa, followed by the FC scaffolds at 0.24 ± 0.08 MPa, and the −20 °C and −80 °C FF scaffolds were 0.17 ± 0.06 MPa and 0.23 ± 0.05 MPa, respectively. There were significant differences between smooth samples and other samples as well as between −20 °C and −80 °C FF scaffolds. The bulk stiffness was lower than wall stiffness for all scaffolds due to the macroscale and microscale pores. The wall stiffness was primarily used for comparison of scaffold properties as it is the stiffness that cells sense. The FF and FC scaffolds had much lower stiffness than smooth scaffolds that could result from the densified polymer network opposed to the swollen hydrogel structure. The smooth and FF scaffolds had bulk stiffness values that were closer together than the wall stiffness values, where the FF scaffolds had considerably lower wall stiffness than smooth scaffolds. The considerable difference between smooth and FF scaffold wall stiffness may be due to differences in the struts: the smooth scaffolds have a swollen hydrogel structure while the FF scaffolds have a compacted polymer structure due to the porous structure and freeze drying, resulting in a difference in the strut cross-sectional area between the scaffold types. The −20 °C FF scaffolds had greater resilience in bending than smooth scaffolds (Figure 5). The FF scaffolds could be bent nearly 180° while the smooth scaffolds could only be bent approximately 90° with a similar applied bending force. Both smooth and FF scaffolds remained intact without damage after bending. When bent further, the smooth scaffolds started to delaminate and lose structural integrity. The reduced cross-sectional area in the FF scaffolds may enable this greater resilience. Further studies will investigate the cause of this resilience and how to leverage it for applications. 

### 3.3. FF Technique Supports Printing of Complex Structures

**Figure 6:**
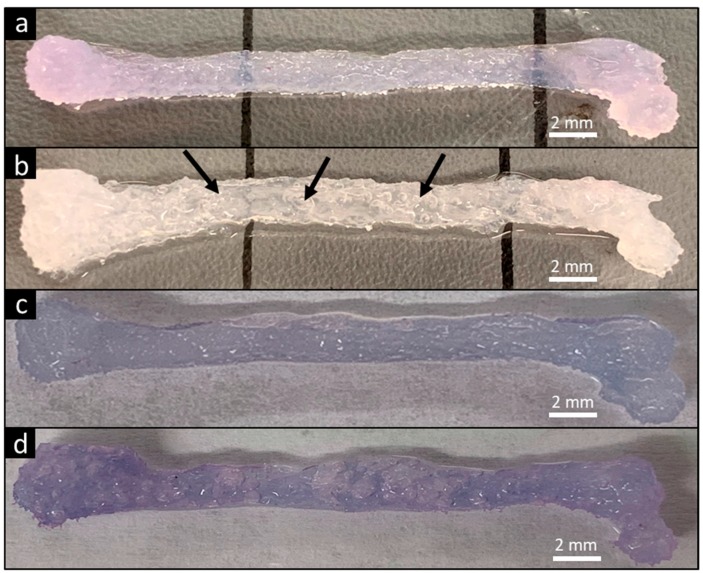
FF technique supports printing of complex designs. A human femur model was used for demonstration. Smooth print (**a**) and FF print (**b**) after recovery from support bath. Pores within the printed FF femur are visible by the presence of entrapped air bubbles (arrows). Immersing the smooth (**c**) and FF prints (**d**) in dye solution caused diffusion of dye throughout the print. The darker color in the FF print demonstrated the formation of interconnected pores in the structure.

The ability of the FF technique to produce complex biological structures was demonstrated by printing a scaled human femur (Figure 6). The FF femur had an opaque color that indicates greater porosity compared to the translucent smooth femur since the pores scatter light (Figure 6a,b). The smooth and FF femurs were immersed in dye solution and the color was compared to evaluate porosity in the printed structure. The FF femurs had a darker color compared to the smooth femurs (Figure 6c,d), confirming the presence of additional porosity in the FF prints.

**Figure 7:**
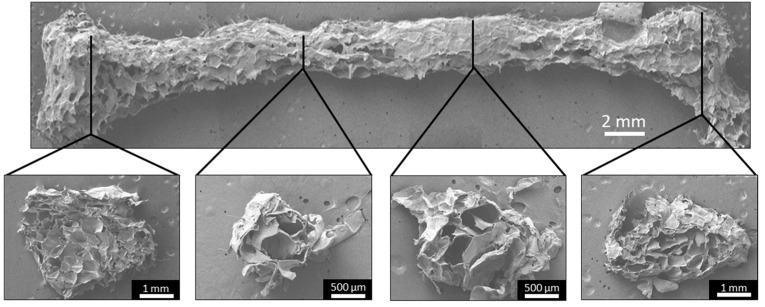
The FF technique creates porosity throughout the printed structure. SEM images of the FF femur display the surface morphology and pore structure. All of the cross-sections had a porous structure, confirming that pore formation occurred throughout the structure.

FF femurs were sectioned at several sites and SEM imaged to analyze the pore structure throughout the print. All of the cross-sections had porous structures, confirming the pore formation throughout the printed structure (Figure 7). However, there was variation in the pore morphologies and pore sizes, possibly due to the material deposition. The femur model is a heterogeneous structure, which caused the printhead to follow contours in the pattern instead of repeating simple transverse movement during printing, resulting in heterogeneous material deposition. Improving the material deposition by altering the printing path may allow for a homogenous pore structure throughout a complex object.

### 3.4. Evaluation of Cell Response to FF Scaffolds

**Figure 8:**
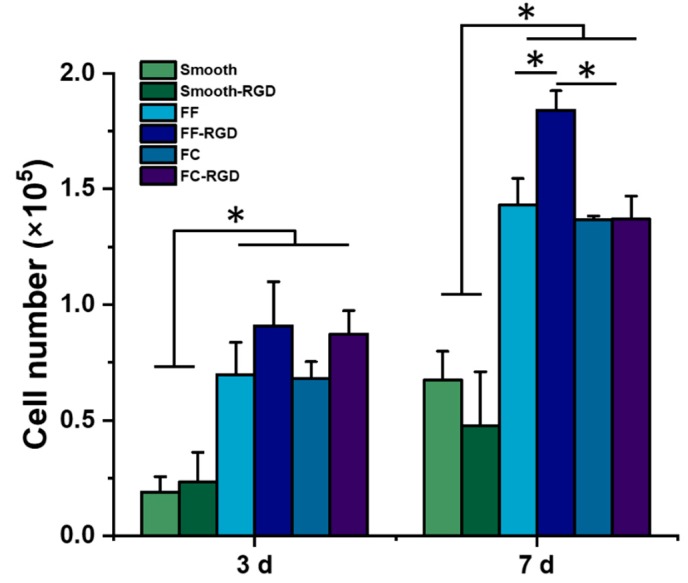
FF scaffolds support breast cancer cell growth. Alginate scaffolds were prepared with and without RGD peptide conjugation as smooth, FF, and FC scaffolds. The scaffolds were evaluated with MDA-MB-231 culture and cell numbers were assayed at 3 d and 7 d. (*) denotes significant difference where *p* < 0.05.

The 231 cells were cultured on FF scaffolds for 7 d to evaluate the cell growth and morphology. The FF scaffolds were prepared with alginate, which has poor cell adhesion due to its negative charge and polysaccharide structure [30], so additional samples for each scaffold type were prepared with RGD-alginate to improve cell adhesion. While the materials properties of −20 °C and −80 °C FF scaffolds were evaluated, only the −20 °C FF scaffolds were evaluated with cell culture since the −20 °C and −80 °C FF scaffolds had similar properties. Cell number on the scaffolds was assessed with the Alamar Blue assay (Figure 8). After 3 d of culture, the FF-RGD and FC-RGD scaffolds had the greatest cell numbers while the smooth and smooth-RGD scaffolds had the lowest cell numbers. The FC scaffolds had similar cell numbers as the corresponding FF scaffolds. Both the FF and FC scaffolds had significantly greater cell numbers than smooth scaffolds. There was no significant difference in cell number for the same scaffold format with and without RGD for all three scaffold formats. The FF and FC scaffolds had significantly greater cell numbers than the smooth scaffolds at 7 d. The FF-RGD scaffolds had the greatest cell numbers, followed closely by the FC-RGD, FF, and FC scaffolds and distantly by the smooth and smooth-RGD scaffolds. All scaffold groups had significantly greater cell numbers at 7 d compared to 3 d except for the smooth-RGD scaffolds.

**Figure 9:**
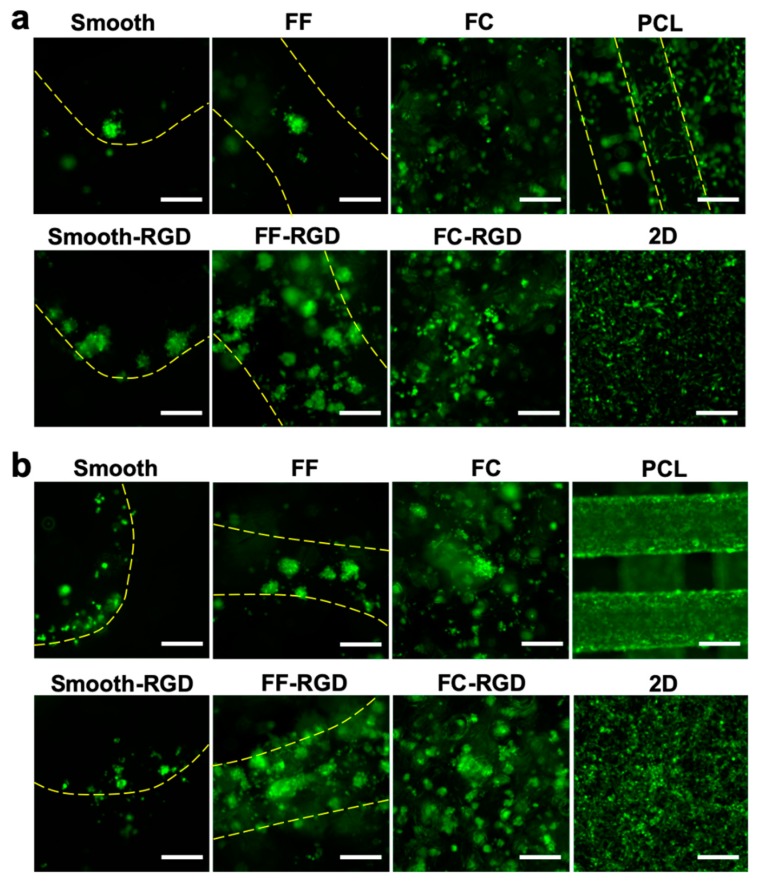
MDA-MB-231 cell distribution on FF scaffolds. Fluorescence images of smooth, FF, FC, and PCL scaffolds and 2D surfaces at 3 d (**a**) and 7 d (**b**). Yellow lines indicate the struts of printed scaffolds. Scale bars are 250 μm.

**Figure 10:**
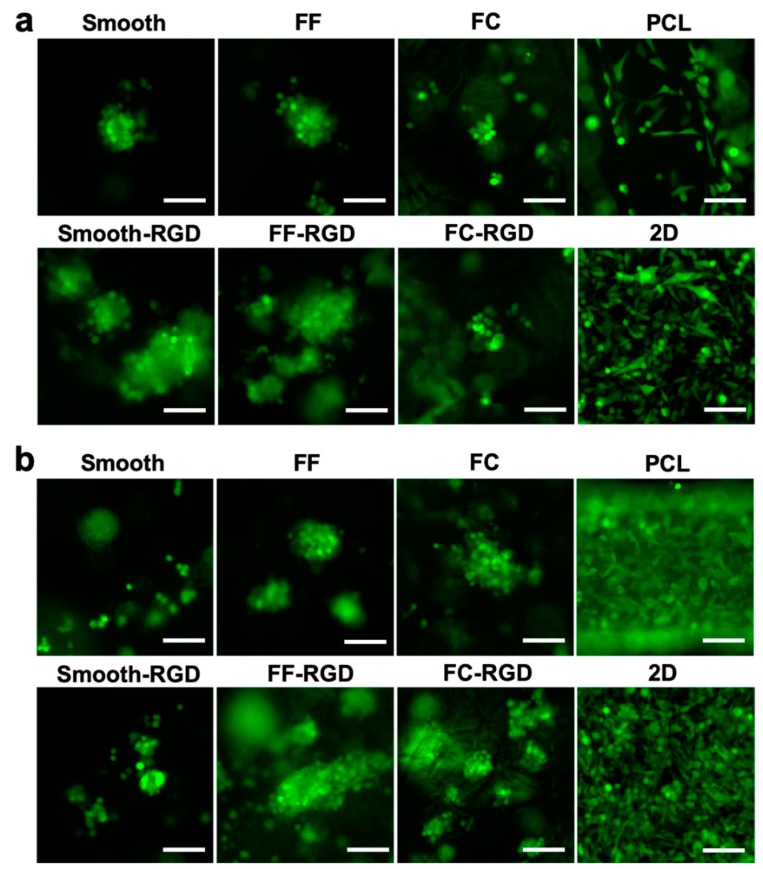
MDA-MB-231 cell morphology on FF scaffolds. Fluorescence images of smooth, FF, FC, and PCL scaffolds and 2D surfaces at 3 d (**a**) and 7 d (**b**). Scale bars are 100 μm.

The 231 cell distribution and morphology on the scaffolds was assessed with live cell fluorescence imaging during the 7 d culture. The 231 cell distribution on the FC, smooth, and FF alginate scaffolds at 3 d is sparse and RGD-alginate enhanced cell adhesion and improved the cell distribution (Figure 9a). Cells were found at the edges of macroscale pores in smooth scaffolds, while cell clusters were found around the struts of FF scaffolds. The PCL scaffold and 2D culture had good cell distribution. The cell distribution improved on the FF, FF-RGD, FC, and FC-RGD scaffolds at 7 d, but decreased on the smooth and smooth-RGD scaffolds (Figure 9b). The 231 cells on PCL scaffolds and 2D had greater cell density after 7 d culture. The 231 cells had a round morphology and aggregated to form multicellular clusters on smooth, smooth-RGD, FF, FF-RGD, FC, and FC-RGD scaffolds, while they had an elongated shape on PCL and 2D at 3 d (Figure 10a). The RGD-alginate did not promote elongated cell morphologies, but it promoted the formation of larger cell clusters. Cells on FC scaffolds had similar morphology as on FF scaffolds. All of the 231 cultures had similar cell morphologies at 7 d as at 3 d, although the smooth scaffolds had fewer and smaller multicellular spheroids (Figure 10b).

Hierarchical porosity promoted greater cell interaction with the printed structure as 231 cells were observed throughout printed struts in the FF scaffolds. The FF scaffolds had greater cell numbers and larger spheroids than the smooth scaffolds. This finding is supported by the literature, as pore size influences cell adhesion and growth, with larger pores promoting greater osteoblast adhesion and growth through increased interaction with the scaffold [36]. While the smooth scaffolds demonstrated poor cell growth, the FRESH technique was developed for bioprinting of cells embedded in the scaffold struts, not for culturing cells seeded on the scaffold, so these results may not be representative of the technique, despite being an appropriate control for the FF scaffolds. There was little change in cell morphology with the increased porosity of the FF scaffolds compared to the smooth and FC scaffolds. This may result from the comparatively low stiffness of the FF, smooth, and FC scaffolds relative to the PCL scaffolds and 2D surface. However, the lower stiffness of the FF, smooth, and FC scaffolds better matches the stiffness of breast tissue than PCL and 2D, which have stiffnesses in a similar range as bone [37]. Additionally, while RGD-alginate samples were prepared, the ligand concentration may have been too low to elicit a change in cell morphology. These issues could be addressed through the development of new polymeric inks with greater stiffness and integrin-binding sites. The development of additional polymeric inks for FF scaffolds will be explored in further studies.

While additional inks may provide greater change in cell morphology with the pores in the FF scaffolds, the multicellular spheroids may be the cellular morphotype [38], or representative cell morphology, for 231 cells in the porous alginate scaffolds at this alginate concentration. Detailed analysis of the cell morphology may reveal subtle differences between the FF, FF-RGD, FC, and FC-RGD scaffold cultures, but it is beyond the scope of this study. The influence of nanoscale and microscale surface features on cell response provided motivation for this work. Cells sense nanoscale and microscale surface features, which influences cell adhesion and promotes differentiation and migration [39,40]. Etched 3DP PCL scaffolds had microscale features and increased surface roughness that induced osteogenic differentiation of human bone marrow stromal cells, while the control 3DP PCL scaffolds did not [9]. The hierarchical structures in the FF scaffolds could be enhanced by incorporating additional features, such as nanoparticles that could be applied to the scaffold surface and influence cell response.

Many biomedical 3DP applications utilize bioprinting, where cells are deposited along with scaffold material [10]. Bioprinting techniques typically encapsulate cells in the printed scaffolds to ensure high seeding efficiency. The cells are added to the polymeric ink and extruded, producing shear stresses that may lead to cell injury and death [41]. Additionally, the bioprinted scaffold surface may limit nutrient and oxygen diffusion from the culture media to cells. The FF technique was developed as a method to produce biomaterial scaffolds with additional features, not a bioprinting technique. Seeding the scaffolds after printing results in reduced cell seeding efficiency compared to bioprinting, but it minimizes the shear stresses on the cells. Additionally, cells seeded onto the FF scaffolds had better contact with the media, providing better nutrition and gas exchange. Therefore, seeding cells onto printed scaffolds may be preferred for maintaining cell vitality compared to bioprinting cells encapsulated in the scaffold, but a comparative study is needed to determine the preferred method.

## 4. Conclusions

We reported the development of Freeze-FRESH, a 3DP technique that incorporates freeze-casting with the FRESH 3DP method to produce scaffolds with microscale pores throughout the scaffold struts. Scaffolds produced with FF method had a hierarchical pore structure due to the macroscale pores in the printed structure and microscale pores in the struts. The −20 °C and −80 °C FF scaffolds had similar pore sizes due to the influence of the support bath on freezing behavior. FF scaffolds had lower bulk and pore wall stiffness than smooth scaffolds but had greater resilience in bending. FF scaffolds had significantly higher numbers of 231 cells than smooth scaffolds over 7 d culture indicating that the microscale pores improved cell growth. The 231 cells formed multicellular clusters on struts of FF scaffolds and had similar morphologies to the cells cultured in the FC scaffolds. Our work demonstrated that FF printing produced scaffolds with hierarchical pore structures that improved breast cancer cell growth. In future studies, other polymer inks will be evaluated for FF printing to broaden the materials that can be used.

## Figures and Tables

**Table 1:** Shrinkage, porosity*, and average pore size of scaffolds.

Sample	Shrinkage (%)	Porosity (%)	Size of Macro-Pores (mm) **	Size of Micro-Pores (µm)
Smooth	73.02 ± 4.44	10.45 ± 2.28	1.26 ± 0.23	N/A
FF (−20 °C)	51.23 ± 6.96	66.67 ± 2.17	1.41 ± 0.26	307.79 ± 97.12
FF (−80 °C)	53.39 ± 7.87	59.63 ± 12.32	1.33 ± 0.25	269.17 ± 58.39
FC	22.06 ± 1.47	90.03 ± 3.31	N/A	152. 42 ± 41.73

*Porosity is strut porosity for smooth and FF scaffolds and bulk porosity for freeze cast (FC) scaffolds. **Macro-pore size of the honeycomb design is 3.20 ± 0.14 mm.
